# Retraction: Hydrogen decreases susceptibility to AngII-induced atrial fibrillation and atrial fibrosis via the NOX4/ROS/NLRP3 and TGF-β1/Smad2/3 signaling pathways

**DOI:** 10.1371/journal.pone.0362511

**Published:** 2026-11-11

**Authors:** The PLOS One Editors

After publication of this article [1], concerns were raised about the results presented in Figs 2 and 5–7. Specifically, when the aspect ratio is altered,

- There appear to be multiple partial and full panel overlaps between panels within Fig 2G, Fig 6A, and Fig 6B of [1].
- There appear to be multiple full panel overlaps between the following figures of [1] and [2–5]:
  - Fig 5B of [1] and Fig 1B of [2].
  - Fig 6C of [1] and Fig 6D, and 6F–J of [3].
  - Fig 7D of [1] and Fig 1A–B of [4].
  - Fig 5C of [1] and Fig 4C of [5].

The first author stated that errors occurred during image selection and figure assembly and noted that there were additional errors in the panel selection for Fig 5A. They shared corrected versions of Figs 2 and 5–7, and provided original triplicate image data underlying Figs 2G, 5A–C, 6A-C, and 7D. Editorial assessment of these data raised further concerns about the integrity and reliability of the underlying dataset. Furthermore, the author’s explanation does not clarify the observed cross-article image overlap.

The first author stated that articles [1] and [5] arose from independent projects prepared during overlapping periods and the Fig 5C images of [1] are incorrect. In light of the above concerns, the *PLOS One* Editors retract this article.

Fig 5B in [1] was previously published in [2] under a CC BY 4.0 license, Fig 6C in [1] was previously published in [3] under a under a CC BY 4.0 license, and Fig 7D in [1] appears similar to content previously published in [4] under a CC BY-NC-ND 4.0 license, without citation of the above articles [2–4] in [1]. Fig 5B, Fig 6C, and Fig 7D are subject to the licenses that apply to the original articles.

All authors agreed with the retraction.
